# Evidence for a Selectively Regulated Prioritization Shift Depending on Walking Situations in Older Adults

**DOI:** 10.3389/fnagi.2017.00075

**Published:** 2017-04-04

**Authors:** Dina Salkovic, Markus A. Hobert, Carolin Bellut, Florian Funer, Sarah Renno, Linda Haertner, Sandra E. Hasmann, Jana Staebler, Johanna Geritz, Ulrike Suenkel, Andreas J. Fallgatter, Gerhard W. Eschweiler, Daniela Berg, Walter Maetzler

**Affiliations:** ^1^Department of Neurodegenerative Diseases, Center for Neurology, Hertie Institute for Clinical Brain Research, University of TuebingenTuebingen, Germany; ^2^German Center for Neurodegenerative DiseasesTuebingen, Germany; ^3^Department of Neurology, Christian-Albrechts-UniversityKiel, Germany; ^4^Department of Psychiatry, University of TuebingenTuebingen, Germany; ^5^Geriatric Center, University of TuebingenTuebingen, Germany

**Keywords:** aging, cognitive flexibility, dual tasking, executive function, gait, prioritization, trail making test

## Abstract

**Background:** Older adults have increased risks of balance issues and falls when walking and performing turns in daily situations. Changes of prioritization during different walking situations associated with dual tasking may contribute to these deficits. The objective of this study was therefore to investigate whether older adults demonstrate changes of prioritization during different walking paths.

**Methods:** In total, 1,054 subjects with an age range from 50 to 83 years were selected from the first follow-up visit of the TREND (Tuebinger evaluation of Risk factors for Early detection of Neurodegenerative Disorders) study. They were classified according to their performance on the Trail Making Test (TMT) into good and poor TMT performers (based on recent results showing that cognitive flexibility affects prioritization strategies during straight walking). Absolute dual-task performance and relative dual-task costs (DTC, relative performance under dual-task conditions compared with single-task conditions) were assessed in two paradigms: walking while subtracting serial 7 s and walking while checking boxes on a clipboard. Both tasks were performed on straight and curved paths.

**Results:** Overall, the poor TMT performers group performed worse in all single and dual tasks. Interestingly, the relative change in performance measured by dual-task costs differed in the groups between the two walking paths. On straight paths, poor TMT performers had a similar DTC of walking to that of good performers (*p* = 0.10) but had a significantly lower DTC of subtracting (*p* = 0.02). On curved paths, poor performers had a similar DTC of subtracting (*p* = 0.10), but their DTC of walking was significantly higher (*p* < 0.0001).

**Conclusion:** Given that walking on curved paths is considered more difficult than that on straight paths and that the serial subtracting dual task is more difficult than the box checking dual task, this study in older adults provides evidence for the existence of a (walking) situation-dependent change of prioritization. If confirmed in other studies, situation-dependent change of prioritization should be included as a potential factor contributing to gait and balance impairments, and increased fall risk in older adults.

## Introduction

Falls are a common problem in older adults (Tinetti et al., 1988; Rubenstein and Josephson, 2002). Especially in this group, falls can lead to serious injuries (Tinetti et al., 1988; Rubenstein and Josephson, 2002) and ultimately even lead to a higher mortality rate (Kannus et al., 2005). Older adults that have experienced a fall are hospitalized more often than non-fallers (Kiel et al., 1991). This emphasizes the need for a detailed assessment that can predict and prevent falls in older adults. In daily life, falls are the result of an interaction of environmental and personal circumstances with risk factors (Bloem et al., 2006; Rubenstein, 2006; WHO, 2007; Yogev-Seligmann et al., 2008). More recently, also the role of cognitive dysfunction as a risk factor for falling was recognized (Woollacott and Shumway-Cook, 2002; Bloem et al., 2006; Holtzer et al., 2006; Yogev-Seligmann et al., 2008; Heinzel et al., 2016). This observation is supported by the overwhelming evidence that there are cognitive demands of gait rather than viewing gait as a series of automated motor actions (Woollacott and Shumway-Cook, 2002; Yogev-Seligmann et al., 2008; Maetzler et al., 2013). Specifically, impaired executive function seems to be a relevant determinant of walking impairment and fall risk in older adults (Binder et al., 1999; Ble et al., 2005; Coppin et al., 2006; Buracchio et al., 2011; Muir-Hunter et al., 2014). This is especially true for patient populations such as patients with Parkinson's disease (PD) (Yogev et al., 2005, 2007; Bloem et al., 2006; Heinzel et al., 2016) and elderly fallers (Springer et al., 2006; Yogev et al., 2007).

To improve knowledge on walking impairment and to understand the cognitive circumstances around falling, research has focused on dual tasking: walking while simultaneously performing a secondary (cognitive) task. Dual tasking forms a large part of daily activities and has been investigated extensively (Pearson, 1993; Guertin, 2013). In dual-tasking paradigms, both the walking as the cognitive task performance can deteriorate when they are compared to the single-task paradigms (Bloem et al., 2006; Yogev-Seligmann et al., 2008). This relative decrease in performance depends on the prioritization strategy that is employed; it indicates which tasks are prioritized during the simultaneous performance of multiple tasks. Over the years, several findings have emerged in this field. It was shown that there are different prioritization strategies depending on the health / disease status, both in adults (Bloem et al., 2001; Rochester et al., 2004) and children (Schott et al., 2016). Moreover, age influences prioritization strategies (Lindenberger et al., 2000; Beurskens and Bock, 2012; Schaefer, 2014), as does cognitive flexibility (Ble et al., 2005; Coppin et al., 2006; Hobert et al., 2011). A study by Lowry found that cognitive flexibility was related to the number of steps performed in a single-task Figure-of-8 Walk Test (F8WT) (Lowry et al., 2012), whereas another study found no associations between cognitive flexibility and the F8WT (Odonkor et al., 2013). Some studies have found that cognitive flexibility is associated with gait speed in dual tasking among older people, especially for walking paths with increasing complexity that are more cognitively demanding (Ble et al., 2005; Coppin et al., 2006; Hirota et al., 2010; Hobert et al., 2011; Killane et al., 2014). Taken together, there is (i) very limited knowledge about the influence of different walking situations on prioritization strategies, and (ii) evidence that cognitive flexibility is associated with prioritization strategies and thus cognitive flexibility seems a promising marker to investigate prioritization.

In this study, we hypothesized that the used prioritization strategy may depend on the situation (straight or curved walking path), and that older adults with poor cognitive flexibility tend to apply more dangerous strategies during difficult walking situations, compared to simple walking situations. We included a curved walking path, as curved walking comprises a large part of the daily route—in some situations even more than straight walking (Glaister et al., 2007). Curved walking poses a computational challenge (Courtine and Schieppati, 2004; Lowry et al., 2012; Odonkor et al., 2013) as it requires a constant asymmetry of trajectory while preserving postural balance, and thus, it involves predictive control of the planned trajectory (Berthoz and Viaud-Delmon, 1999; Courtine and Schieppati, 2003). Also, turning measures are related to fall risk in older adults (Dite and Temple, 2002; Welch et al., 2016).

## Methods

### Ethics

The study was approved by the ethical committee of the Medical Faculty of the University of Tuebingen (Nr. 90/2009BO2), and all participants provided written informed consent.

### Participants

The TREND study (Tuebinger evaluation of Risk factors for Early detection of Neurodegenerative Disorders, http://www.trend-studie.de) is a longitudinal cohort study that includes participants with and without risk factors for Parkinson's and Alzheimer's disease (e.g., hyposmia, depression, REM sleep behavior disorder). The first follow-up of the study took place in 2011 and 2012 and comprised 1,102 participants aged 50–83 years who were prospectively investigated. Detailed descriptions of the TREND study design, including inclusion and exclusion criteria and baseline assessments, are reported elsewhere (Gaenslen et al., 2014).

For this analysis, we used the cross-sectional data of the 1,102 participants from the first follow-up. Among them, a total of 48 participants were excluded from analysis due to the following reasons: 12 met the criteria for Parkinson's disease according to the UK Brain Bank Society criteria, 10 were physically unable to complete the movement assessment, 11 had incomplete data, three had negative delta Trail Making Test (TMT) values, 11 had a Mini-Mental Score Examination score <25, and one was in an alcoholic condition during assessment. Finally, a total of 1,054 participants were included in the analysis. Their demographic and basic clinical characteristics are provided in Table 1.

**Table 1:** **Demographics and clinical assessments and trail making test performance**.

	**Good TMT performers (*n* = 348)**	**Poor TMT performers (*n* = 346)**	**Whole cohort (*n* = 1054)**	***p*-value**	***t*-value**	**Degrees of freedom**	**Cohen's *d***
Male [%] ^*^	50.0	54.3	51.7	0.25	1.31	1	0.043
Age [years]	63 ± 7	67 ± 7	65 ± 7	<0.0001	−6.3	1	0.47
Body height [m]	1.71 ± 0.09	1.70 ± 0.08	1.71 ± 0.09	0.08	1.4	1	0.10
Body weight [kg]	75 ± 14	77 ± 14	76 ± 14	0.17	−1.8	1	0.13
BMI	25.5 ± 3.9	26.4 ± 4.2	26.0 ± 4.0	0.0028	−3.0	1	0.23
Grip force [kg]	31.8 ± 10.3	31.1 ± 10.6	31.5 ± 10.3	0.43	−0.8	1	0.06
Education period [years]	15 ± 3	14 ± 3	14 ± 3	<0.0001	6.5	1	0.49
BDI (0–63)	6 ± 6	7 ± 6	6 ± 6	0.12	−1.6	1	0.12
MMSE	29 ± 1	28 ± 1	28 ± 1	<0.0001	6.6	1	0.50
TMT A [s]	36 ± 12	42 ± 14	38 ± 13	<0.0001	−6.3	1	0.48
TMT B [s]	60 ± 13	126 ± 43	89 ± 38	<0.0001	−27.0	1	2.1
Delta TMT [s]	24 ± 7	84 ± 36	50 ± 33	<0.0001	−30.3	1	2.3
TUG [s]	9.8 ± 2.5	10.5 ± 2.8	10.0 ± 2.4	0.0014	−3.2	1	0.24

Good performers were defined as having a delta Trail Making Test (TMT) score of <35 s, poor performers as having a delta TMT score higher than 54 s. Intermediate performers (defined as having a delta TMT score of 35–54 s) are not shown. Data are presented with mean and standard deviation. Comparisons of good and poor performers group were done using t-test and ^*^ Chi square test. Results are presented using p-values, t-values (^*^ F-value), degrees of freedom and Cohen's d (^*^ phi). BDI, Beck's Depression Inventory; BMI, Body Mass Index; MMSE, Mini-Mental State Examination; TUG, Timed Up and Go test.

### Task conditions

All participants performed the tasks in the same order. First of all, there were two single non-walking tasks: subtracting and checking boxes (Bock and Beurskens, 2011; Beurskens and Bock, 2012). The participants performed the subtracting task with maximum speed, where they had to subtract serial 7 s from a three-digit number until 10 subtractions were completed. The instruction was as follows: “Please subtract serial 7 s as fast as you can from the number I will shortly tell you, until I interrupt you.” This was followed by the checking boxes task. During this task, the participants held a clipboard with a sheet of paper in their non-dominant hand and a pen in their other hand. They were asked to mark a cross within each of the 32 boxes on the sheet of paper with a pencil. The instruction was as follows: “Please mark each of the boxes on the sheet of paper with a cross as fast as you can.”

Subsequently, the participants performed several tasks on a CWP. The curved walking trial was defined as walking three times (i.e., 1,080°) around a marked circle on the floor with a diameter of 1.2 m. Walks in the clockwise and counterclockwise directions were alternated to avoid the effects of direction. Participants started with the single walking trial [task (i)] by walking three times in a counterclockwise direction, followed by three times in a clockwise direction. This was followed by a dual task [task (ii)] in which participants walked while checking boxes, starting with three times in a counterclockwise direction and again followed by three times in a clockwise direction. The second dual task [task (iii)] consisted of walking while subtracting serial 7 s. For this task, a three-digit number was told to the participant directly before the start sign was given (a different number than previously used). No hint regarding the prioritization of any task was given to omit an external influence on the prioritization process (Yogev-Seligmann et al., 2010). Participants performed these tasks three times in a counterclockwise direction, followed by three times in a clockwise direction. The instructions for walking on the curved walking path were “Please walk three times clockwise/counterclockwise around the marked circle with convenient gait speed and do not risk falling” for task (i), “Please walk three times clockwise / counterclockwise around the marked circle with convenient gait speed and do not risk falling, and mark each of the boxes on the sheet of paper with a cross as fast as you can” for task (ii), and “Please walk three times clockwise / counterclockwise around the marked circle with convenient gait speed and do not risk falling, and subtract serial 7 s as fast as you can from the number I will shortly tell you” for task (iii). The total walking distance of walking on a CWP comprises approximately 14 m.

Finally, tasks were performed on a SWP, for which the starting and ending locations were marked on the floor. The single walking task [task (i)] consisted of walking 20 m until the marked end of the path in a 150-cm-wide corridor, allowing for an obstacle-free walk. The first dual task [task (ii)] consisted of walking while checking boxes once on a SWP, and the second dual task [task (iii)] consisted of walking while subtracting with another three-digit number to start with. The instructions for walking on the straight walking path were “Please walk as fast as you can, do not run, do not risk falling “for task (i), “Please walk as fast as you can, do not run, do not risk falling, and mark each of the boxes on the sheet of paper with a cross as fast as you can” for task (ii), and “Please walk as fast as you can, do not run, do not risk falling, and subtract serial 7 s as fast as you can from the number I will shortly tell you” for task (iii).

The examiner documented the time it took to perform a task (measured with a stopwatch) as well as the number of checked boxes, the number of subtractions, and the number of subtraction errors. In all tasks, the performance of checking boxes was measured by calculating the time it took, on average, to check one box. The performance of subtracting was measured by calculating the time it took, on average, for one subtraction. For walking on a CWP, the time for one walking trial was measured (the speed could not be calculated, because the walking distance is not exactly determined) whereas for walking on an SWP, the speed of one walking trial was measured.

### Cognitive assessment

To evaluate cognitive flexibility, the Trail Making Test (TMT) was used (Reitan, 1958; Corrigan and Hinkeldey, 1987; Arbuthnott and Frank, 2000; Sánchez-Cubillo et al., 2009). In part A of the TMT, the participants draw lines on a sheet of paper to connect randomly spread numbers (from 1 to 25) in an ascending numerical order as fast as possible. In part B, the participants connect randomly spread numbers (from 1 to 13) and letters (from A to L) alternatingly in an ascending order (i.e., 1-A-2-B-3-C-…-13-L). The direct score of each part is represented by the completion time, and TMT performance is calculated by subtracting the time needed for TMT-A from the time needed for TMT-B. This delta TMT value “removes” eventual bias due to differences in upper extremity motor speed, simple sequencing, visual scanning, and psychomotor functioning (Reitan, 1958; Corrigan and Hinkeldey, 1987; Arbuthnott and Frank, 2000; Ble et al., 2005; Sánchez-Cubillo et al., 2009). In the case of an error, the examiner draws the attention of the participant to the error so that it can be corrected; however, this correction is included in the completion time.

### Categorization into groups

Participants with delta TMT values >54 s were defined as poor performers (lowest tertile, *n* = 346), those with delta TMT values ranging from 35 to 54 s as intermediate performers (*n* = 360), and those with delta TMT values <35 s as good performers (highest tertile, *n* = 348).

### Data processing and statistical analysis

Data were analyzed with JMP software (version 11.1.1, SAS) and are presented as the mean value and standard deviation if not otherwise indicated. To compare the demographic and basic clinical variables of the between good and poor TMT performer groups, the *t*-test (or, in case of categorical data, the chi square test) was used (Table 1). We did not include the group of intermediate TMT performers, as the objective of the study did not focus on the effect of cognitive flexibility *per se*, but on differences between cohorts regarding walking situation dependency of prioritization. The calculated differences are group differences in each task, which are used to describe a difference of prioritization change between the two cohorts depending on the task situation (e.g., straight and curved walking path). The outcome variables (Tables 2, 3) were calculated with a logistic regression model, corrected for age, gender, body mass index, education level, and the Mini-Mental State Examination score. Significance of each model effect was assessed by the likelihood ratio. Differences were considered significant at *p* < 0.05 (two-sided). For the SWP, the walking speed was calculated in the single- and dual-task conditions. For the CWP, the walking time was calculated using the mean value of the walks in the clockwise and counterclockwise directions. The parameters “box-checking speed” and “subtracting speed” were respectively defined as the number of checked boxes or subtractions over the time needed for the task (seconds). As a next step, dual-task costs (DTC) were determined, reflecting the relative performance in the dual-task setting as compared to the single task setting. They were calculated using the following formula according to Bock (2008) and Lindemann et al. (2010): DTC [%] = (single task-dual task) / (single task) ^*^ 100. This formula gives information about the percentage change relative to the single-task value. A positive DTC value indicates a decrease in the performance during the dual-task condition compared to the single-task condition, and vice versa. The parameter “subtraction errors” was defined as the proportion of people within a cohort who made at least one subtraction error. Eventually, situation dependency of the prioritization strategy was determined by visually comparing significance levels between cohorts during the two tasks, SWP and CWP.

**Table 2:** **Single and dual-task results**.

	**Good TMT performers**	**Poor TMT performers**	***p*-value**	***F*-value**	**Degrees of freedom**	**Effect size**	***R*^2^**	**Odd's ratio (95% CI)**
**SINGLE TASKS**								
Walking speed on SWP [m/s]	1.67 ± 0.24	1.56 ± 0.25	0.077	3.13	1	0.003	0.35	0.5 (0.2–1.1)
Walking time on CWP [s]	16.0 ± 0.9	16.8 ± 3.3	0.95	0.004	1	0.000	0.20	1.0 (0.9–1.1)
Checking boxes [1/s]	1.64 ± 0.26	1.45 ± 0.26	<0.0001	37.43	1	0.040	0.28	0.1 (0.1–0.2)
Subtracting serial 7 s [1/s]	0.47 ± 0.17	0.34 ± 0.15	<0.0001	74.72	1	0.087	0.22	0.0 (0.003–0.03)
≥1 subtraction error (proportion of cohort) [%]	33	56	<0.0001	28.23	1	0.039	0.08	2.4 (1.7–3.4)
**DUAL-TASK CONDITIONS—SWP**								
Walking when checking boxes [m/s]	1.48 ± 0.23	1.38 ± 0.23	0.18	1.79	1	0.002	0.30	0.6 (0.3–1.3)
Checking boxes when walking [1/s]	1.42 ± 0.27	1.25 ± 0.27	<0.0001	23.74	1	0.026	0.26	0.04 (0.01–0.14)
Walking when subtracting [m/s]	1.40 ± 0.27	1.27 ± 0.25	0.003	8.81	1	0.010	0.26	0.3 (0.2–0.7)
Subtracting when walking [1/s]	0.44 ± 0.17	0.34 ± 0.14	<0.0001	50.39	1	0.064	0.16	0.02 (0.01–0.07)
≥1 subtraction error (proportion of cohort) [%]	35	46	0.039	4.29	1	0.006	0.03	1.4 (1.0–2.0)
**DUAL-TASK CONDITIONS—CWP**								
Walking when checking boxes [s]	19.3 ± 4.3	20.3 ± 5.0	0.79	0.07	1	0.000	0.21	1.0 (1.0–1.0)
Checking boxes when walking [1/s]	1.40 ± 0.25	1.23 ± 0.25	<0.0001	28.32	1	0.031	0.27	0.04 (0.01–0.1)
Walking when subtracting [s]	19.4 ± 5.1	21.7 ± 5.5	0.0013	10.46	1	0.014	0.13	1.1 (1.0–1.1)
Subtracting when walking [1/s]	0.45 ± 0.16	0.34 ± 0.13	<0.0001	73.80	1	0.088	0.20	0.01 (0.0–0.2)
≥1 subtraction error (proportion of cohort) [%]	31	47	0.0013	10.38	1	0.015	0.03	1.7 (1.2–2.4)

*Data are presented with mean and standard deviation. P-Values, F-Values, degrees of freedom, effect size (eta square), R-squared values, and Odd's ratios were calculated using a logistical regression model and the likelihood ratio, with correction for age, gender, body mass index, education level, and the Mini-Mental State Examination score. CWP, circular walking path; SWP, straight walking path; TMT, Trail Making Test. Note that the parameters for walking on the SWP are displayed with walking speed [m/s] and parameters for walking on the CWP with time needed to walk three times around a circle with 1.2 m diameter [s]*.

**Table 3:** **Dual-task costs**.

	**Good TMT performers**	**Poor TMT performers**	***p*-value**	***F*-value**	**Degrees of freedom**	**Effect size**	***R*^2^**	**Odd's ratio (95% CI)**
**DUAL-TASK COSTS—SWP**								
Walking when checking boxes [%]	11 ± 8	11 ± 9	0.61	0.26	1	0.000	0.05	0.6 (0.1–4.1)
Checking boxes when walking [%]	13 ± 11	14 ± 12	0.80	0.06	1	0.000	0.02	0.8 (0.2–3.3)
Walking when subtracting [%]	16 ± 11	18 ± 12	0.10	2.76	1	0.004	0.03	3.2 (0.8–12.8)
Subtracting when walking [%]	3 ± 28	−4 ± 37	0.023	5.23	1	0.008	0.02	0.5 (0.3–0.9)
≥1 subtraction error (proportion of cohort) [%]	2	−10	0.014	6.08	1	0.009	0.02	1.4 (1.1–1.8)
**DUAL-TASK COSTS —CWP**								
Walking when checking boxes [%]	20 ± 16	21 ± 17	0.67	0.18	1	0.000	0.08	0.8 (0.3–2.3)
Checking boxes when walking [%]	15 ± 10	15 ± 11	0.96	0.003	1	0.000	0.04	1.0 (0.2–4.9)
Walking when subtracting [%]	22 ± 24	30 ± 25	<0.0001	19.14	1	0.028	0.03	4.5 (2.2–9.4)
Subtracting when walking [%]	2 ± 22	−3 ± 35	0.10	2.66	1	0.004	0.03	0.6 (0.3–1.1)
≥1 subtraction error (proportion of cohort) [%]	−2	−10	0.11	2.51	1	0.004	0.02	1.2 (1.0–1.6)

*Data are presented with mean and standard deviation. P-Values, F-Values, degrees of freedom, effect size (eta square), R-squared values, and Odd's ratios were calculated using a logistical regression model and the likelihood ratio, with correction for age, gender, body mass index, education level, and the Mini-Mental State Examination score. CWP, circular walking path; SWP, straight walking path*.

## Results

Under single-task conditions, there were no statistically significant group differences in walking performance on either the SWP or the CWP. However, significant group differences were found in the box-checking speed, the serial 7 subtracting speed, and the number of performers with at least one subtraction error. Details are provided in Table 2.

Dual tasks that were performed on a SWP revealed group differences similar to dual tasks that were performed on a CWP (Table 2). When the participants walked while checking boxes, on both the SWP and the CWP, there were no statistically significant group differences in walking performance, while the box-checking speed was significantly lower for the poor TMT performers. When the participants walked while subtracting, the groups differed significantly in their performances. For the poor TMT group, the speed of walking while subtracting as well as subtracting while walking was significantly lower compared to the good TMT group. Also, the number of performers with at least one subtraction error was significantly higher in the poor TMT group. This was the case on both the SWP and the CWP.

**Figure 1:**
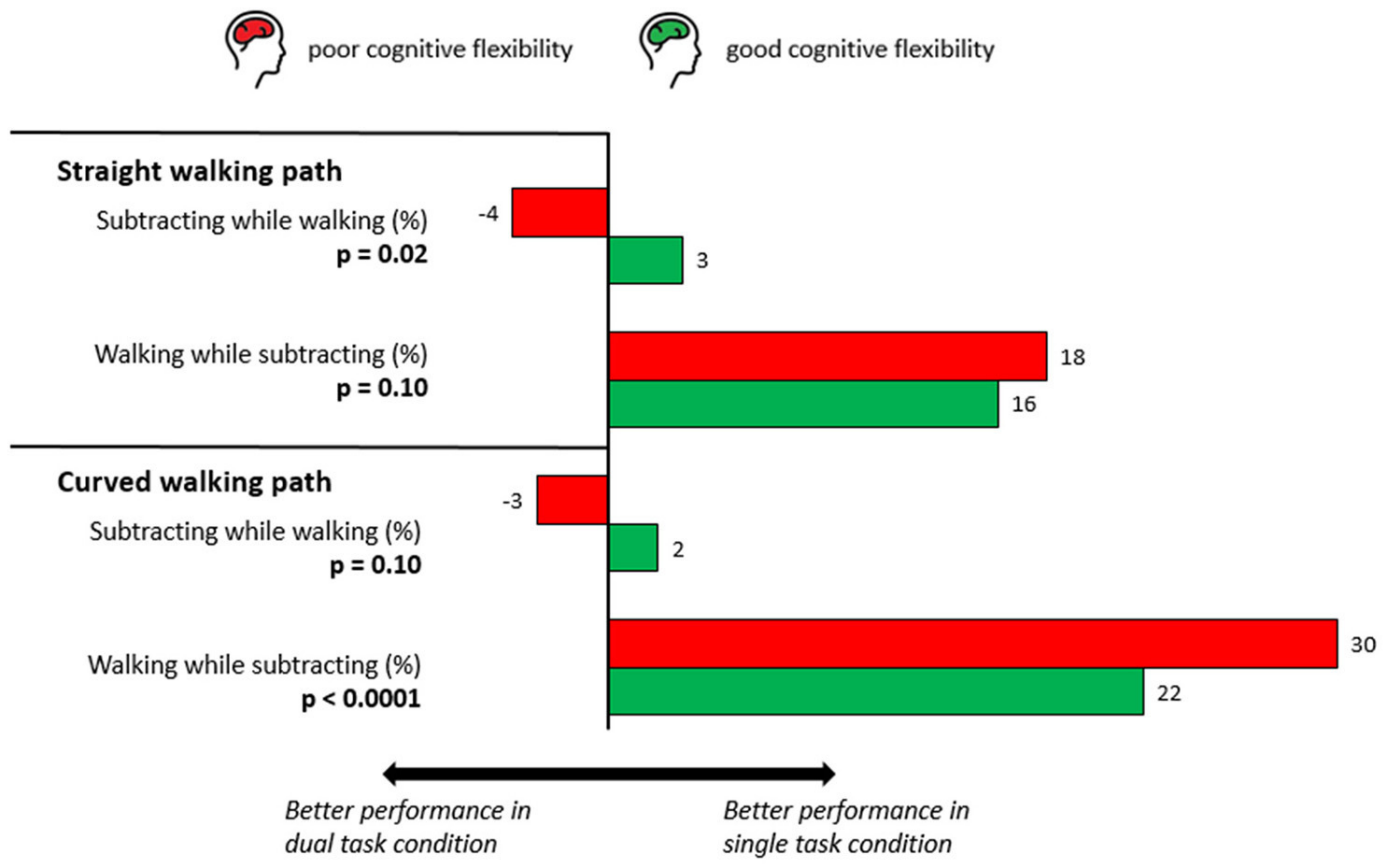
**Dual-task costs (DTC, see data processing and statistical analysis for details) of subtracting while walking on a straight (SWP) and a curved walking path (CWP)**. Note the different patterns of significance for the respective situations and the paths. In the SWP, older adults with poor cognitive flexibility did not significantly differ in their DTC with respect to walking performance but reached a significantly lower DTC value for the subtraction task. In the CWP situation, the significance pattern is converse, indicating a change in prioritization.

The TMT groups did not significantly differ in the DTC of walking while checking boxes on either one of the walking paths, neither in walking performance nor in box-checking speed. However, the DTC of walking while subtracting revealed specific differences between the good and poor TMT performers. Although the DTC of subtracting speed and the proportion of people who had at least one subtraction error significantly differed between the groups on the SWP, these parameters did not differ significantly on the CWP. In contrast, the DTC of walking speed was significantly different between the groups on the CWP but not on the SWP. The DTC results are provided in Table 3, Figure 1.

## Discussion

In an effort to analyze the situation dependency of prioritization strategies of older people under different task conditions, we evaluated the DTC of walking and non-walking tasks on a SWP and a CWP. Our data suggest that there are indeed differences in the weighting of the two simultaneously performed tasks according to cognitive flexibility and the walking situation (CWP vs. SWP).

Not surprisingly, for the SWP, the groups differed significantly in their performance of non-walking single tasks and in their dual-task performance of walking while subtracting. These results support previous studies in the field (Ble et al., 2005; Coppin et al., 2006; Hirota et al., 2010; Hobert et al., 2011; Killane et al., 2014) Accordingly, for the CWP, the absolute values of single- and dual-task performances revealed group differences similar to the observed differences on SWP.

The focus of this study was on the comparison of change in prioritization strategies between tasks performed on the SWP and CWP by looking at the DTC. Indeed, there was a contrasting finding in DTC of the groups. On the SWP, the DTC of walking while subtracting did not differ, while the DTC of subtracting while walking did significantly differ between the two groups. On the CWP, the opposite was true: the DTC of walking while subtracting did differ, while the DTC of subtracting while walking did not significantly differ between the groups (Figure 1). The number of participants that made subtraction errors also suggests that people with poor cognitive flexibility demonstrate more dangerous dual-task strategies in challenging walking situations. Although not statistically significant (*p* = 0.11), the DTC of subtraction errors among the poor TMT performers during curved walking was −10% (i.e., a *decrease* of 10 percent in the number of participants that made subtraction errors in the dual-task condition compared to the single-task condition). The DTC of subtraction errors was only 2.0% in the good TMT performers. Taken together, these data suggest a focus on subtracting rather than walking in the dual task in the poor TMT performers relative to the good TMT performers during the more complex walking task (or, vice versa, a focus of the good TMT performers on walking rather than subtracting). This argues in favor of our hypothesis that prioritization is indeed influenced by (walking) situations. Our results indicate that different walking situations in cohorts with poor cognitive flexibility lead to a prioritization shift that is different from cohorts with good cognitive flexibility. This can be considered, from a gait and balance point of view, as dangerous. The consideration of such prioritization changes in future treatment and prevention strategies may provide further insights into the influence of this factor on balance impairment and falls in the elderly. We also believe that our results do not oppose the posture first strategy mentioned in previous research of healthy older adults (Bloem et al., 2006; which in fact reports about the typical behavior during straight walking).

How can our finding be integrated in the existing pathomechanistic aspects of dual tasking? Dual tasks require additional resources, such as dividing attention and visual navigation, which makes these tasks in fact multi-task assessments. Here, the dual tasks we focused on were walking while subtracting (a motor task and a cognitive task) and walking while checking boxes (two motor tasks; Hobert et al., 2011). There were no differences in walking DTC while checking boxes on either one of the paths. Therefore, this task did not demonstrate different prioritization strategies, suggesting that the simultaneous performance of two motor tasks (i.e., walking and checking boxes) and a further cognitive task (dividing attention) is insufficiently challenging to delineate relevant differences between the groups. In contrast, simultaneously performing one motor task (walking) and two cognitive tasks (subtracting and dividing attention) may be more challenging for older adults, and it is thus capable of showing different decays in performance between two cohorts with different cognitive flexibility function, as is shown by our results. This dual-task interference may be best explained by the “capacity sharing” model of dual-task interference (Tombu and Jolicœur, 2003), which states that tasks can be performed in parallel but there are limited resources allocated among the tasks. The central “bottleneck” model (Welford, 1952) which states that when two tasks are performed, the initial processing of one task is delayed until the processing of the first task is completed, may not perfectly explain our results.

### Limitations

This study has some limitations. The first one lies in the task design. In the dual tasks on an SWP, the participants were instructed to walk as fast as possible, whereas participants walked at a convenient speed during the dual tasks on a CWP. We hypothesized that walking at maximum speed on a curved path would cause unnecessary stress (Yogev-Seligmann et al., 2008) and subsequently lead to an increased risk of falling and a decrease in performance that could not have been entirely attributed to cognitive flexibility. However, in our view, this difference does not challenge our main results as all of the groups had an identical study protocol. Second, we chose a turning diameter of approximately 150 cm (participants walked around a circle with a diameter of 120 cm). A longer or more difficult path might also reveal more differences between the groups or provide us with task performance that more closely resembles daily situations. However, turning in the home-like environment consists of few steps with limited duration (El-Gohary et al., 2013), so we feel that the approach used here for turning assessment is a good compromise between scientific accuracy and daily relevance. Third, we must take into account that a possible increase in walking time might be due to a longer walkway that was used by the participants for walking on CWT. However, such behavior would also indicate the existence of a different prioritization shift when walking and simultaneously performing a second task. Fourth, we analyzed data from a single assessment, whereas longitudinal assessments would perhaps provide us with additional information on the described differences. Fifth, effect sizes are small. However, we know that many well-established treatment strategies in medicine have low effect sizes (Leucht et al., 2015), and that such approaches can have large effects on an individual level. Additionally, navigational strategies are most probably involved in our tasks and may influence dual-task performance and even prioritization in certain challenging walking tasks (Bock and Beurskens, 2011; Beurskens and Bock, 2012) but the assessment of navigational aspects during our experiments was beyond the scope of our study.

### Conclusion

This study demonstrates that prioritization in older adults is influenced by the (walking) situation. Older adults with poor cognitive flexibility tend to risky walking behavior during more complex walking situations, compared to older adults with good cognitive flexibility. Our results provide new insights into fall circumstances and fall risk in older adults and suggest that the influence of prioritization aspects during turning strategies should be investigated in more depth.

## Author contributions

DS, MH, DB, and WM made substantial contributions to the acquisition, analysis, and interpretation of data for the work. CB, FF, SR, LH, SH, JS, JG, US, AF, and GE made substantial contributions to the acquisition of the data. DS, MH, and WM drafted the paper, all remaining authors revised the draft critically for important intellectual content. All authors gave their final approval of the version to be published, and agree to be accountable for all aspects of the work in ensuring that questions related to the accuracy or integrity of any part of the work are appropriately investigated and resolved.

## Funding

MH received travel grants by Abbvie and Merz. DB is member of an Advisory Board of UCB pharma GmbH and receives honoraria from UCB pharma GmbH. She reports grants from Michael J. Fox Foundation, Janssen Pharmaceutica N.V., German Parkinson's Disease Association (dPV), BMWi, BMBF, Parkinson Fonds Deutschland gGmbH, UCB Pharma GmbH, TEVA Pharma GmbH, EU, Novartis Pharma GmbH, Boehringer Ingelheim Pharma GmbH, Lundbeck. WM serves on the editorial board of PLOS ONE, received funding from the European Union, the Michael J. Fox Foundation, Robert Bosch Foundation, Neuroalliance, Lundbeck and Janssen, and holds part of a patent for the assessment of dyskinesias (German patent office, 102015220741.2). He received speaker honoraria from GlaxoSmithKline, Abbvie, UCB, Licher MT, and Rölke Pharma.

### Conflict of interest statement

The reviewer KW declared a shared affiliation, though no other collaboration, with several of the authors MH, JG, DB and WM to the handling Editor, who ensured that the process nevertheless met the standards of a fair and objective review. The reviewer KW declared a past co-authorship with one of the authors DB to the handling Editor, who ensured that the process met the standards of a fair and objective review. The other authors declare that the research was conducted in the absence of any commercial or financial relationships that could be construed as a potential conflict of interest.

## Abbreviations

CWP: Curved walking path
SWP: Straight walking path
TMT: Trail Making Test
DTC: Dual-Task Cost.
